# Phthalates and Childhood Asthma: Revealing an Association through Urinary Biomarkers

**DOI:** 10.1289/ehp.121-a59

**Published:** 2013-02-01

**Authors:** Tanya Tillett

**Affiliations:** Tanya Tillett, MA, of Durham, NC, is a staff writer/editor for *EHP*. She has been on the *EHP* staff since 2000 and has represented the journal at national and international conferences.

Exposure to phthalates, substances used as plasticizers in a large number of consumer goods, can occur by ingestion, inhalation, or skin contact. Children––who are uniquely vulnerable to adverse health effects of environmental exposures because of their still-developing neuro-logical, immunological, and respiratory systems—can receive particularly high exposures due to more frequent contact with phthalate-rich surfaces such as plastic toys and polyvinyl chloride (PVC) flooring, as well as house dust, which collects phthalates (and other chemicals). Researchers now report an association between phthalate exposure and asthma and allergic disease in a cohort of 10-year-old Norwegian children [*EHP* 121(2):251–256; Bertelsen et al.].

The research team conducted a cross-sectional analysis of urine sample data from 623 children participating in the 10-year followup to the prospective Environment and Childhood Asthma study in Oslo. The followup included structured interviews with parents and blood tests, lung function tests, clinical examinations, and skin prick tests for allergic sensitization for children. Samples of first-morning urine were collected and assessed for three metabolites of low-molecular-weight phthalates (commonly used in personal care products) and eight metabolites of high-molecular-weight phthalates (commonly used in PVC applications such as flooring, electrical cords, car interiors, and toys). The investigators used logistic regression modeling to estimate associations between current asthma and phthalate metabolite concentrations.

**Figure:**
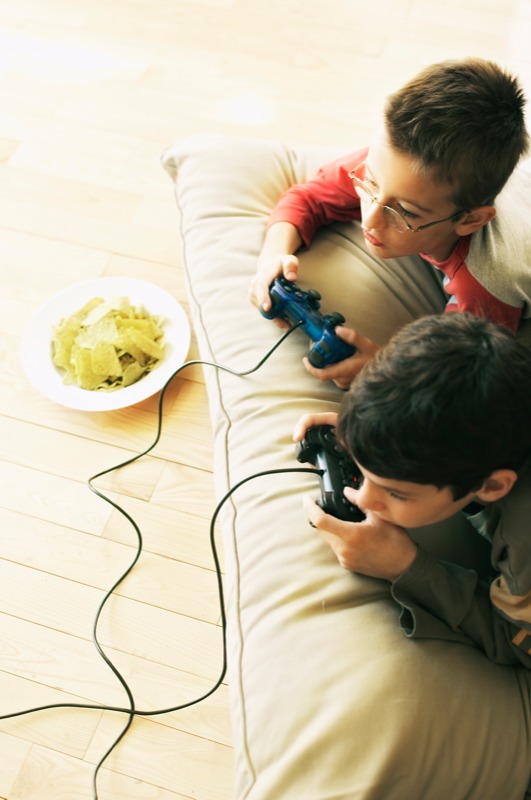
Hand-to-mouth activity combined with phthalate-rich surfaces such as PVC toys and dusty floors can mean particularly high phthalate exposures for children. © Ken Kaminesky/Take 2 Productions/Corbis

Previous animal studies have shown an association between high-molecular-weight phthalates and allergic disease development, while epidemiological studies have shown exposure to house dust containing several of these chemicals to be associated with asthma and allergic disease. The current analysis revealed an association between asthma in children and the highest quartiles of exposure to the metabolites mono-carboxyoctyl phthalate and mono-carboxynonyl phthalate. The parent compounds of these metabolites are high-molecular-weight phthalates used primarily as plasticizers in PVC items. No association was observed between asthma and the nine other metabolites, nor was any association detected between any metabolites and allergic sensitization.

Limitations of the study include the cross-sectional design and measurements based on single urine samples, which do not reflect phthalate exposures over time. However, the findings do support the growing body of evidence showing an association between asthma and exposure to high-molecular-weight phthalates.

