# Erratum: “Phthalates and Childhood Asthma: Revealing an Association through Urinary Biomarkers”

**DOI:** 10.1289/ehp.121-a239

**Published:** 2013-08-01

The February 2013 News article “Phthalates and Childhood Asthma: Revealing an Association through Urinary Biomarkers” [Environ Health Perspect 121:A59 (2013)] referred to plastic/polyvinyl chloride toys as “phthalate-rich” in a discussion of phthalate exposures among 10-year-old Norwegian children. The Consumer Product Safety Improvement Act of 2008 and European Commission Decision C(1999) 4436 of 7 December 1999 eliminated or suspended the use of certain phthalates of concern in toys and children’s products. The children discussed in the News article were assessed in 2001–2004 and may have had access to toys purchased before the 1999 European ban. However, it was incorrect to suggest that all plastic toys continue to be “rich” sources of phthalates, and the article has been revised accordingly.

*EHP* regrets the error.

